# Pea Ferritin Stability under Gastric pH Conditions Determines the Mechanism of Iron Uptake in Caco-2 Cells

**DOI:** 10.1093/jn/nxy096

**Published:** 2018-06-22

**Authors:** Antonio Perfecto, Ildefonso Rodriguez-Ramiro, Jorge Rodriguez-Celma, Paul Sharp, Janneke Balk, Susan Fairweather-Tait

**Affiliations:** 1 Norwich Medical School, University of East Anglia, Norwich, United Kingdom; 2 School of Biological Sciences, University of East Anglia, Norwich, United Kingdom; 3 Department of Biological Chemistry, John Innes Center, Norwich, United Kingdom; 4 Diabetes and Nutritional Sciences Division, King's College London, London, United Kingdom

**Keywords:** pea ferritin, phytoferritin, DMT-1, iron absorption, bioavailability, endocytosis

## Abstract

**Background:**

Iron deficiency is an enduring global health problem that requires new remedial approaches. Iron absorption from soybean-derived ferritin, an ∼550-kDa iron storage protein, is comparable to bioavailable ferrous sulfate (FeSO_4_). However, the absorption of ferritin is reported to involve an endocytic mechanism, independent of divalent metal ion transporter 1 (DMT-1), the transporter for nonheme iron.

**Objective:**

Our overall aim was to examine the potential of purified ferritin from peas (*Pisum sativum*) as a food supplement by measuring its stability under gastric pH treatment and the mechanisms of iron uptake into Caco-2 cells.

**Methods:**

Caco-2 cells were treated with native or gastric pH–treated pea ferritin in combination with dietary modulators of nonheme iron uptake, small interfering RNA targeting DMT-1, or chemical inhibitors of endocytosis. Cellular ferritin formation, a surrogate measure of iron uptake, and internalization of pea ferritin with the use of specific antibodies were measured. The production of reactive oxygen species (ROS) in response to equimolar concentrations of native pea ferritin and FeSO_4_ was also compared.

**Results:**

Pea ferritin exposed to gastric pH treatment was degraded, and the released iron was transported into Caco-2 cells by DMT-1. Inhibitors of DMT-1 and nonheme iron absorption reduced iron uptake by 26–40%. Conversely, in the absence of gastric pH treatment, the iron uptake of native pea ferritin was unaffected by inhibitors of nonheme iron absorption, and the protein was observed to be internalized in Caco-2 cells. Chlorpromazine (clathrin-mediated endocytosis inhibitor) reduced the native pea ferritin content within cells by ∼30%, which confirmed that the native pea ferritin was transported into cells via a clathrin-mediated endocytic pathway. In addition, 60% less ROS production resulted from native pea ferritin in comparison to FeSO_4_.

**Conclusion:**

With consideration that nonheme dietary inhibitors display no effect on iron uptake and the low oxidative potential relative to FeSO_4_, intact pea ferritin appears to be a promising iron supplement.

## Introduction

Iron deficiency is the most prevalent nutritional deficiency in the world, affecting 1–2 billion people worldwide (1, 2). Plant-based foods represent the main source of dietary iron for the majority of the world's population, but their iron bioavailability is low (1–10%) (3, 4) in comparison to meat, mainly due to the presence of phytates and polyphenols, which bind nonheme iron in the intestinal lumen, preventing absorption (4–7). Identifying sources of iron from plant foods with high bioavailability represents a strategic priority given the growing pressure to reduce meat consumption in relation to lowering the risk of chronic diseases, improving food sustainability, and slowing global warming.

A form of bioavailable iron identified in plants is phytoferritin (plant ferritin), which is found in high concentrations in peas, beans, soybeans, and other pulses (6, 8, 9). Plant ferritin is a heteropolymeric 24-subunit complex surrounding an iron core, which can store ≤4500 iron atoms in the form of ferric oxyhydroxide-phosphate (8). Plant ferritin is composed of 2 types of subunits, H-1 and H-2, which have been shown to be involved in different functions related to the oxidative deposition of iron (10). However, the ratio of these 2 protein subunits is plant species dependent (8, 11). This may explain the variation in total iron bound to plant ferritin, which has been reported to vary considerably between different legumes, ranging from 18% in soybeans to 42% in dry peas (12). There is evidence that iron from soybean ferritin is as well absorbed as ferrous sulfate (FeSO_4_) (6, 13), but it is unclear whether ferritin is degraded in the gastrointestinal tract and the iron subsequently released (9, 12, 14), or whether it is absorbed intact through an endocytosis-like pathway (15, 16). The latter route of uptake could confer an advantage because the protection of iron by the protein coat obstructs binding with dietary inhibitors of nonheme iron absorption (17). In addition, despite the fact that pea ferritin sequesters more than twice the total iron found in soybean ferritins (12), to our knowledge pea ferritin has not been investigated in the context of human iron nutrition.

The aim of this study was to explore the potential of purified ferritin from peas as a source of iron in foods and food supplements. In addition, we also compared the cellular redox status of Caco-2 cells exposed to either native pea ferritin or FeSO_4_ as a predictor of gastrointestinal tolerance when consumed as a food supplement.

## Methods

### Reagents

All chemicals, unless otherwise stated, were obtained from Sigma-Aldrich.

### Pea ferritin purification

Dried peas, *Pisum sativum* cv. Sakura (sold as “marrowfat” peas in the United Kingdom) were donated by Wherry and Sons (Rippingale, United Kingdom) and used for pea ferritin extraction. The purification procedure was similar to the method of Laulhere et al. (18), with an added gel filtration purification step using a Superose 12 10/300 GL column (GE Healthcare) with PBS as the running buffer. The amount of protein was determined by using the Bradford method (BioRad), and iron was quantified by using the colorimetric iron chelator Ferene-S [3-(2-pyridyl)-5,6-di(2-furyl)-1,2,4-triazine-5′,5′′-disulfonic acid]. The purified ferritin was also used to raise rabbit polyclonal antibodies (Covalab).

### Pea ferritin stability at different gastrointestinal pHs

To assess the stability of pea ferritin at an adult (pH 2) and an infant (pH 4) gastric pH, a modification of the procedure of Hoppler et al. (12) was used. Briefly, pea ferritin or ferric ammonium citrate (FAC) was added to 140 mM NaCl and 5 mM KCl pH 2 or pH 4 (with 0.1 M HCl) and incubated for 1 h at 37°C. After 1 h, the solutions were readjusted to pH 6.9–7.0 with 1 M NaHCO_3_ and incubated for 30 min. The solutions were diluted in MEM and incubated with cells for 1 or 24 h. Alternatively, for comparison of protein stability and cellular uptake at neutral pH, the native form of pea ferritin was directly diluted in MEM (pH 7).

### Cell culture

Caco-2 cells (HTB-37 VA) were obtained from the American Type Culture Collection. Cells were cultured in flasks containing DMEM (Gibco) supplemented with 10% FBS, 1% nonessential amino acid solution, 1% penicillin/streptomycin, and 1% l-glutamine and incubated in 5% CO_2_/95% air atmosphere at constant humidity. The media were replaced every 2–3 d. Cells were seeded onto collagen-coated 6-, 12-, 24-, or 96-well plates (depending on experiment) and were grown to postconfluence for 12 d. Experiments were conducted with the use of cell passages 25–40. In order to ensure low basal media iron concentrations, cells were replaced with MEM 24 h before iron treatment, as previously described (19).

### Cell treatments

To evaluate the mechanisms of nonheme iron uptake from native and gastric pH–treated pea ferritin, the iron absorption enhancer ascorbic acid (AA; molar ratio of Fe:AA: 1:20), an Fe^2+^ iron chelator [bathophenanthroline disulfide (BPDS); 50 µM] and an iron absorption inhibitor of divalent metal ion transporter 1 (DMT-1)–mediated iron uptake [calcium chloride (CaCl_2_); 2.5 mM)] were used. Cells were treated with AA, BPDS, or CaCl_2_ for 24 h together with (native or gastric pH–treated) pea ferritin or FAC at 30 µM Fe.

Endocytosis pathways in Caco-2 cells from native and gastric pH–treated pea ferritin were examined by incubating cells with chlorpromazine hydrochloride (CPZ; 100 µM) and sucrose (0.5 M) to inhibit clathrin-mediated endocytosis, as previously described (15, 20). In order to inhibit caveolae-mediated endocytosis and macropinocytosis, filipin (8 µM) and dimethyl amiloride (200 µM) were used, respectively, as previously described (15, 21). For these experiments, cells were co-incubated for 1 h with the different endocytosis inhibitors in combination with native or gastric pH–treated pea ferritin (100 µM Fe).

### Iron uptake in Caco-2 cells

Cellular ferritin formation (nanograms of cell ferritin per milligram of cell protein) was used as the surrogate marker of iron uptake. Cells were lysed with 200 µL CelLytic protein extraction buffer (Sigma) containing protease inhibitor cocktail (Roche) and centrifuged (at room temperature 14,000 × *g* for 15 min), and the supernatants collected and analyzed for cell ferritin and total cell protein with the use of the Spectroferritin ELISA kit (Ramco) and the BCA protein assay kit (ThermoFisher Scientific). Ferritin concentrations were normalized to total cell protein and based on the percentage from FAC. Negative controls (without added iron) were included in each experiment.

### Cell viability

The CellTiter 96 Aqueous One Solution colorimetric assay (Promega) was used to determine cell viability. Briefly, Caco-2 cells were seeded in 96-well plates as previously described. Pea ferritin or FAC containing 30–500 µM Fe was treated with cells for 24 h. After 24 h, the treatments were removed and replaced with MTS solution diluted in MEM, and the cells were incubated for 3 h. The absorbance of each well was measured by using a microplate reader at 490 nm.

### Western blotting

The concentrations of pea ferritin protein (0.1 mg/mL) at pH 2 and 4 were monitored for the indicated times by Western blot. The effect of endocytosis inhibitors on native pea ferritin uptake (40 nM protein) in Caco-2 cells after 1 h was also measured by Western blot. Equal volumes (20 µL) of gastric pH–treated pea ferritin (at the indicated times and pH) or equal amounts of cellular protein (40 µg) were diluted in 4× loading buffer, incubated for 5 min (95°C), loaded separately onto 15% acrylamide gels, and run at 150 V for 1 h. Separated proteins were transferred to a nitrocellulose membrane and incubated with antibodies raised against purified pea ferritin (1:5000) diluted in Tris-buffered saline with 0.1% (vol:vol) Tween-20 and 5% (wt:vol) skimmed milk, followed by incubation with secondary antibody (goat anti-rabbit, HRP conjugate). Immunoreactive bands were visualized with the use of enhanced chemiluminescence reagents and imaged (ImageQuant LAS 500; GE Healthcare). Signal intensities were quantified with the use of ImageJ software (developed at NIH).

### Total, soluble, and core iron determination

The iron phases from gastric pH–treated pea ferritin were determined by using methods similar to Pereira et al. (20). Aliquots were ultrafiltered (3 kDa molecular weight cut-off (MWCO), Vivaspin 500; GE Life Sciences) at 15,000 × *g* (10 min). Iron concentrations from the ultrafiltered iron and total iron were determined by using Ferene-S. A non-ultrafiltered sample served as a reference for total iron. Solubilized iron released from pea ferritin was determined as the ratio between the iron concentration of the filtrate and total iron. The iron concentration of the core was estimated as follows:

(1) \begin{eqnarray*} {\rm Fe}_{\rm core} &=& {\rm Fe}_{\rm total} - {\rm Fe}_{\rm soluble} - {\rm Fe}_{\rm protein} \nonumber\\ & & \times\, ( \hbox{\% of control observed in the Western blots}) \end{eqnarray*}

### Small interfering RNA knockdown of SLC11A2 in Caco-2 and Hutu-80 cells

Caco-2 cells were seeded in collagen-coated 12-well plates (200,000 cells/well) for 10 d. Cell monolayers were transfected by using Lipofectamine 3000 with Silencer Select small interfering RNA (siRNA) targeting solute carrier family 11 member 2 (*SLC11A2*) (DMT-1; 200 nM; Life Technologies) in Opti-MEM (Gibco) for 48 h. After 48 h, siRNA complexes were removed and replaced with iron treatments for 2 h. The iron treatments were replaced with MEM for a further 22 h. Cells were extracted and harvested for functional uptake assays (total protein, ferritin ELISA, or Western blot) or for RNA extraction and RT-PCR.

Hutu-80 cells, a human epithelial adenocarcinoma adherent cell line obtained from the American Type Culture Collection, were also used for siRNA transfections. Hutu-80 cells were seeded in 12-well plates (100,000 cells/well) until 50–70% confluent. Cell monolayers were transfected with Silencer Select siRNA targeting *SLC11A2* (10 nM; Life Technologies) in Opti-MEM for 48 h. Iron treatments and incubation times were similar to Caco-2 transfections.

### RNA extraction and RT-PCR

Total RNA was extracted from cells with the use of the RNeasy Mini Kit (Qiagen) according to the manufacturer's instructions. RNA quality was determined by using the UV-Vis Nanodrop 2000 spectrophotometer. RNA was reverse transcribed by using the qPCRBIO cDNA Synthesis Kit (PCR Biosystems). Relative qPCR was performed with the use of 4 µL cDNA, SYBR Green Mix Lo-ROX (PCR Biosystems), and the following predesigned primers (KiCqStart SYBR Green Primers, Sigma)—*SLC11A2*: forward, GAG TAT GTT ACA GTG AAA CCC; reverse, GAC TTG ACT AAG GCA GAA TG; *18S*: forward, ATC GGG GAT TGC AAT TAT TC; reverse, CTC ACT AAA CCA TCC AAT CG. After preamplification (95°C, 2 min), the PCR was amplified for 45 cycles (95°C, 15 s; 58°C, 10 s; 72°C, 10 s) using the LightCycler 480 instrument (Roche). The relative expression of *SLC11A2* was normalized to the housekeeping gene *18S* and assessed with the use of the 2-∆∆Ct method (22).

### Reactive oxygen species generation in Caco-2 cells

Reactive oxygen species (ROS) generation was determined by using the dichlorofluorescin-diacetate assay (23) with minor modifications. Caco-2 cells were seeded in 24-well plates as previously described. Cells were treated with 10 µM dichlorofluorescin-diacetate for 30 min at 37°C. Cells were washed with PBS, and pea ferritin or FeSO_4_ (100 µM Fe) was added. Free-radical generation was measured over time (2 h) with the use of an excitation of 485 nm and an emission of 530 nm.

### Statistical analysis

Data are presented as mean values ± SEMs unless otherwise indicated. When data were not normally distributed, log transformation was used before the statistical analysis. Two-factor ANOVA with Tukey's multiple comparisons test was used to evaluate differences in iron uptake between pea ferritin and FAC when >2 variables were studied together. One-factor ANOVA with Dunnett's multiple comparisons test was used to compare differences in cell ferritin concentrations when cells were incubated with pea ferritin and endocytosis inhibitors. Differences were considered significant at *P* < 0.05. Statistical analysis was performed with the use of GraphPad Prism version 6.0.

## Results

Pea ferritin was extracted from dried peas (*P. sativum*) and purified to homogeneity (**Supplemental Figure 1A**). The iron content was 2360 ± 20 Fe atoms (*n* = 3 purifications) based on a calculated molecular mass of 552 kDa of the protein 24-mer. Antibodies raised against pea ferritin displayed high specificity, with a limit of detection at 0.5 ng pea ferritin (Supplemental Figure 1B). Pea ferritin did not cross-react with the human ferritin ELISA kit used as the marker for iron uptake (**Supplemental Figure 2**). Pea ferritin and FAC treatments did not affect Caco-2 cellular viability at the doses used (**Supplemental Figure 3**).

**FIGURE 1:**
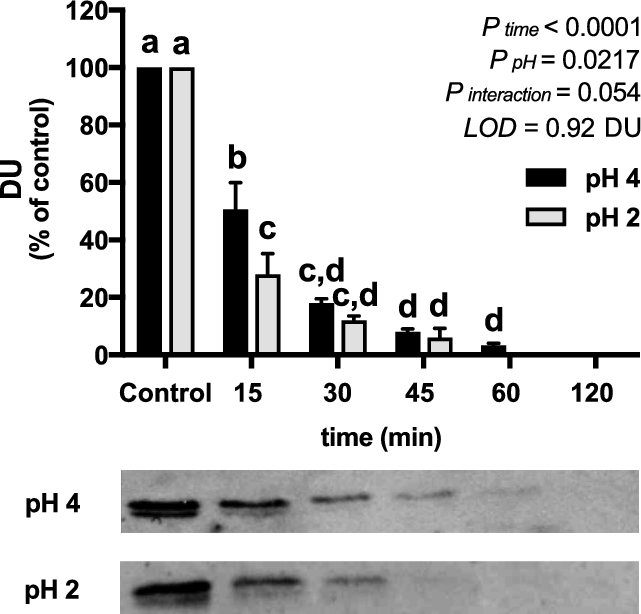
Effect of time on release of iron from gastric pH-treated pea ferritin; LOD = 0.92 DU. Values are means ± SEMs, *n* = 3 independent experiments. Labeled means without a common letter differ, *P* < 0.05. DU, densitometry units; LOD, limit of detection.

The stability of pea ferritin at gastric pH was first assessed. Pea ferritin was not stable at pH 2 or 4, but the rate of degradation was faster at the lower pH; after 15 min at pH 2, 70% was degraded compared with 50% at pH 4 (**Figure 1**). In addition, pea ferritin was fully degraded in the presence of pepsin at pH 2 at 15 min (**Supplemental Figure 4**). At pH 2, without pepsin, the concentrations of intact ferritin were below the limit of detection at 60 min. Under these same conditions, soluble iron released from the ferritin core increased over time; at pH 2, 80% of iron was solubilized after 120 min compared with 25% at pH 4 (**Supplemental Figure 5A, B**).

**FIGURE 2:**
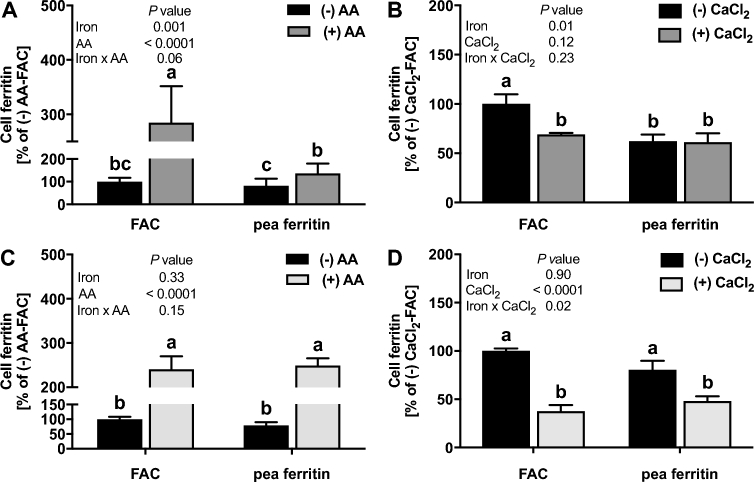
Effect of a nonheme iron promoter (AA; A, C) or inhibitor (CaCl_2_; B, D) on iron uptake in Caco-2 cells exposed to native (A, B) or gastric pH–treated (C, D) pea ferritin or FAC for 24 h. Values are means ± SEMs, *n* = 3 independent experiments. Labeled means without a common letter differ, *P* < 0.05. AA, ascorbic acid; FAC, ferric ammonium citrate.

**FIGURE 3:**
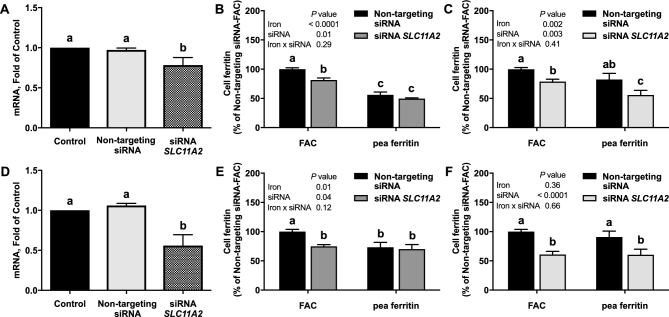
Effect of siRNA knockdown of DMT-1 on iron uptake in Caco-2 (B, C) or Hutu-80 cells (E, F) cells exposed to native (B, E) or gastric pH–treated (C, F) pea ferritin or FAC for 1 h. *SLC11A2* knockdown efficiency in Caco-2 (A) and Hutu-80 (D) cells. Values are means ± SEMs, *n* = 3 independent experiments. Labeled means without a common letter differ, *P* < 0.05. DMT-1, divalent metal ion transporter 1; FAC, ferric ammonium citrate; siRNA, small interfering RNA; *SLC11A2*, solute carrier family 11 member 2.

Next, we measured iron uptake from pea ferritin. Caco-2 cells exposed to FAC at 30 µM Fe had a mean ± SEM value of 26.2 ± 1.7 ng ferritin/mg cell protein, whereas blanks had a value of 5.4 ± 0.7 ng ferritin/mg cell protein. The addition of AA increased iron uptake from both native and gastric pH–treated pea ferritin. The increase was more pronounced in gastric pH–treated ferritin and was similar to FAC (**Figure 2A, C**). CaCl_2_ decreased iron uptake from gastric pH–treated pea ferritin but had no effect on iron uptake from native pea ferritin (Figure 2B, D). Similarly, iron uptake into Caco-2 cells was inhibited when gastric pH–treated pea ferritin was incubated with BPDS for 24 h, but the Fe^2+^ chelator had no effect on native pea ferritin (**Supplemental Figure 6A, B**). To investigate this further, we used siRNA knockdown to target *SLC11A2* gene expression, the gene encoding DMT-1, in Caco-2 and Hutu-80 cells (**Figure 3A, D**). No effect of DMT-1 knockdown on iron uptake from native pea ferritin in both intestinal cell lines was observed (Figure 3B, E). However, iron uptake from FAC and gastric pH–treated pea ferritin was significantly decreased (Figure 3C, F). Collectively, these data clearly show that iron uptake from native pea ferritin occurs independently from DMT-1.

**FIGURE 4:**
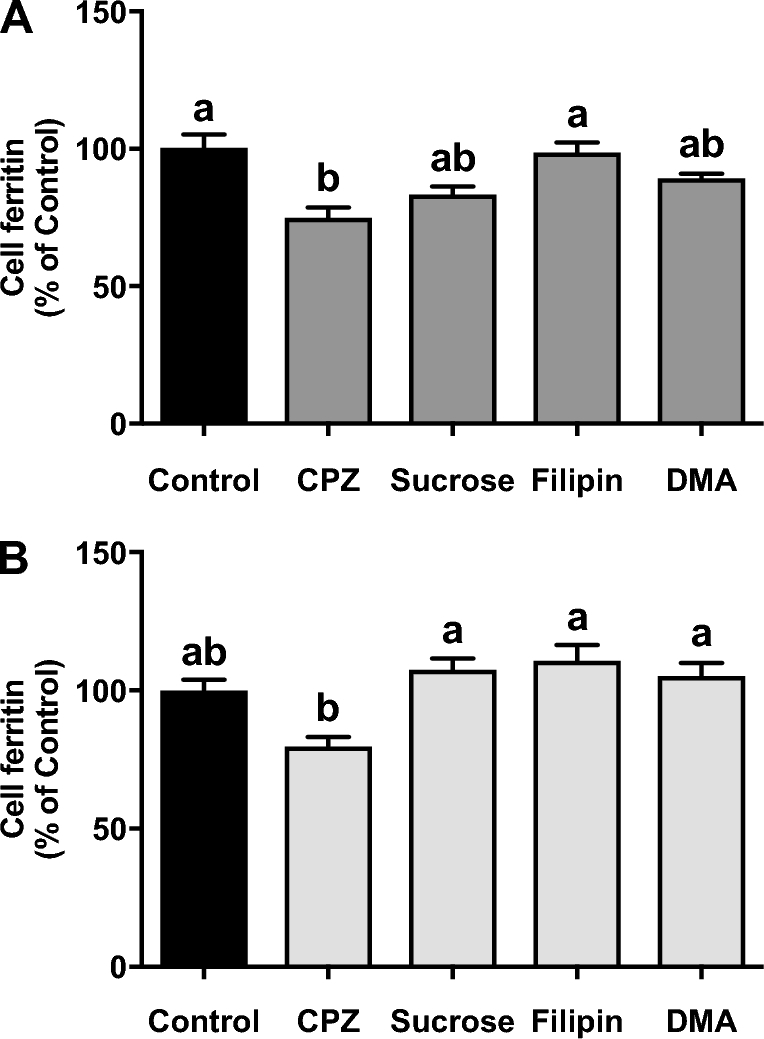
Effect of endocytosis inhibitors on iron uptake in Caco-2 cells exposed to native (A) or gastric pH–treated (B) pea ferritin for 1 h. Values are means ± SEMs, *n* = 3 independent experiments. *Different from control, *P* < 0.05. CPZ, chlorpromazine hydrochloride; DMA, 5-(N,N-Dimethyl)amiloride hydrochloride.

**FIGURE 5:**
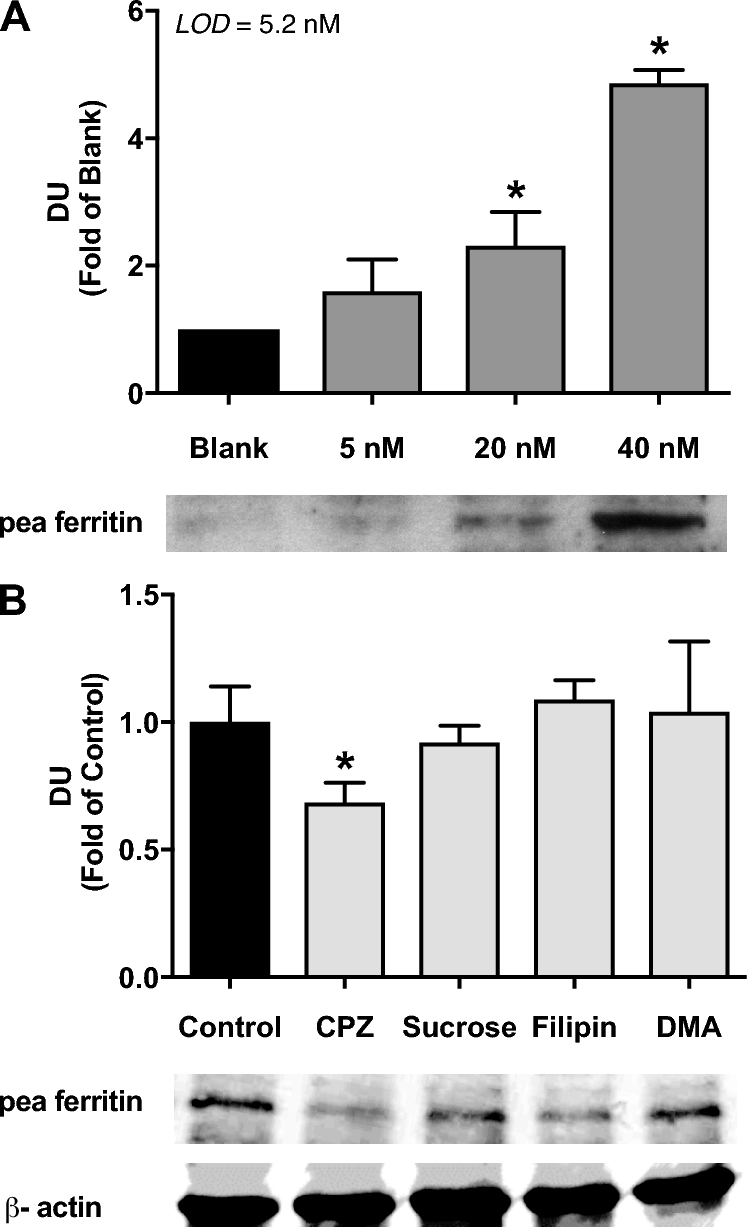
Intracellular detection of pea ferritin in Caco-2 cells exposed to native ferritin (5, 20, and 40 nM protein (A) or native ferritin (40 nM) and endocytosis inhibitors (B) for 1 h. LOD = 5.2 nM. Values are means ± SEMs, *n* = 3 independent experiments. *Different from control, *P* < 0.05. CPZ, chlorpromazine hydrochloride; DMA, 5-(N,N-Dimethyl)amiloride hydrochloride; DU, densitometry units; LOD, limit of detection.

The clathrin endocytosis inhibitors, CPZ and sucrose, significantly decreased iron uptake from native pea ferritin (**Figure 4A**) but did not significantly affect iron uptake from gastric pH–treated pea ferritin (Figure 4B). In contrast, the inhibition of caveolae-mediated endocytosis or macropinocytosis had no effect on iron uptake from native or gastric pH–treated pea ferritin (Figure 4). To confirm uptake of native pea ferritin in Caco-2 cells, cell extracts were analyzed by Western blotting. A significant increase in the detection of the pea ferritin protein taken up by Caco-2 cells was detected when cells were subjected to 20 and 40 nM of native pea ferritin (**Figure 5A**). With the use of the same chemical inhibitors targeting endocytosis, only CPZ inhibited pea ferritin uptake in Caco-2 cells (Figure 5B).

**FIGURE 6:**
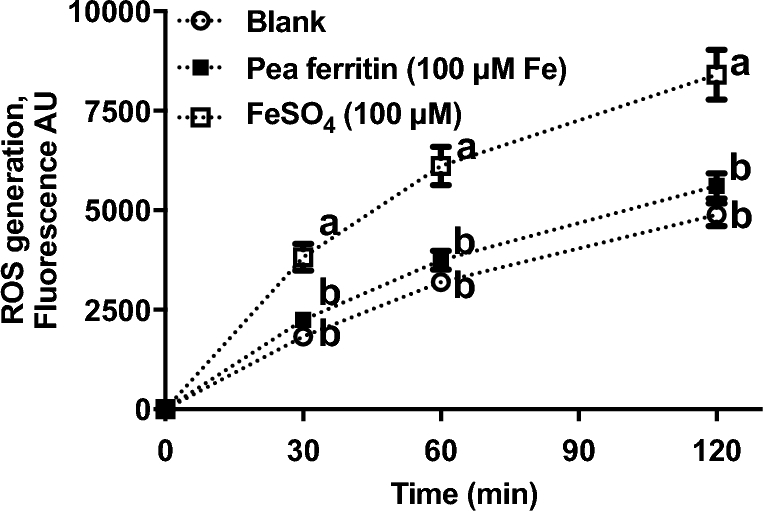
ROS generation in Caco-2 cells exposed to pea ferritin or FeSO_4_ for 0, 30, 60, and 120 min. Values are means ± SEMs, *n* = 3 independent experiments. Means at a time without a common letter differ, *P* < 0.05. AU, arbitrary units; ROS, reactive oxygen species.

ROS generation after 30 min of iron treatment was 60% higher with FeSO_4_ compared with pea ferritin (**Figure 6**). The difference in ROS generation was sustained over time, for ≤2 h, indicating that iron delivered by pea ferritin resulted in lower cellular redox imbalance than that by FeSO_4_.

## Discussion

The overall aim of this study was to evaluate the potential of pea ferritin as an iron supplement, making use of a cost-effective method developed for ferritin extraction and purification from marrowfat peas. We measured the stability of pea ferritin under gastric pH conditions and found that the mechanism for iron uptake into Caco-2 cells was dependent on whether ferritin was degraded or remained intact.

There is disagreement as to whether plant ferritin is fully degraded during passage through the stomach. Theil et al. (16) reported that soybean ferritin remains mainly intact during gastric and luminal digestion, whereas Hoppler et al. (12) observed complete degradation at pH 2 and Lv et al. (24) observed partial degradation at a less acidic pH (pH 3–5). We found that at pH 2, pea ferritin was clearly unstable, and we also observed that pea ferritin exposed to pH 2 and pepsin resulted in rapid degradation after 15 min (Supplemental Figure 4), which agrees with the results of Jin et al. (25). Protein degradation was accompanied by an increase in the percentage of iron released into the medium, and was related to time and lower pH, which supports the observations made by Hoppler et al. (12). The unique characteristics of proteins from different pulses (26), the methods of purification, and conditions of digestion may also have an impact on the stability of plant ferritin and could help explain the different findings between studies.

Three possible mechanisms for iron uptake from plant ferritin have been proposed: full hydrolysis of iron to Fe^2+^ and Fe^3+^ and subsequent Fe^2+^ uptake via DMT-1, Fe^3+^ core uptake, and plant ferritin protein/receptor-bound uptake (27). In our experiments, we found that pea ferritin exposed to a low pH resulted in complete protein degradation and led to the significant release of soluble iron from the iron core. In Caco-2 cells, a well-characterized intestinal cell line to study nutrient absorption and transport (28), we observed that incubation with native or gastric pH–treated pea ferritin with AA increased iron uptake. The increase in iron uptake when pea ferritin was fully degraded is likely related to the solubilizing properties of AA (29). One possible explanation for AA increasing iron uptake from native pea ferritin, where the majority of the iron is present in the core, is the mobilization of iron that occurs naturally in plant physiology in the presence of reductants, as previously described by other authors (30, 31). This indicates that in the presence of dietary factors such as AA, there is an alternative mechanism for iron uptake from pea ferritin that does not involve full degradation of the protein or endocytic mechanisms. In addition, when we blocked cellular DMT-1–mediated iron transport using different approaches, we found that iron uptake was only inhibited with gastric pH–treated pea ferritin. We found an interactive effect on the cellular ferritin formation between iron treatment (FAC compared with pea ferritin) and CaCl_2_ or BPDS, but only when pea ferritin was pH-treated (Figure 2D, Supplemental Figure 6B). Because CaCl_2_ and BPDS are inhibiting iron uptake via DMT-1, this result suggests that only the iron from pH-treated pea ferritin is taken up via DMT-1, whereas a different mechanism is utilized for native pea ferritin. This is in agreement with Kalgaonkar and Lonnerdal (17) who reported a reduction in cellular iron uptake from digested plant ferritin in the presence of dietary factors such as phytates and tannins, presumably by the chelation of free iron.

There are distinct structural differences between animal and plant ferritins (8), which may affect the rate of intestinal iron uptake and bioavailability (24). Plant ferritin, specifically, is composed of 2 subunits, H-1 and H-2, and the ratio between these varies between plants (32, 33). In particular, pea ferritin contains more H-2 subunits than soybean ferritin (32, 33). Lv et al. (24) observed that when recombinant H-1 and H-2 subunits from soybean ferritin were reassembled at different ratios, the rate of binding to the cellular surface and ferritin uptake significantly differed, suggesting the importance of ferritin subunit composition on the rate of uptake by endocytosis. In particular, soybean and pea ferritin differ by ∼20% in their amino acid sequence (33, 34), thus raising the question as to whether this difference in structure could alter the binding and subsequent uptake of pea ferritin by different endocytic mechanisms. To address this question, we used endocytosis inhibitors targeting different uptake mechanisms, including clathrin- and caveolae-mediated endocytosis, and macropinocytosis. We observed that only CPZ reduced the cellular uptake of native pea ferritin, indicating a clathrin-mediated specificity, which has been previously described by San Martin et al. (15) for soybean ferritin. This confirms that both pea and soybean native ferritin are taken up by cells via a similar endocytic mechanism. It is important to note that we also observed a minor decrease in iron uptake (nonsignificant) when gastric pH–treated pea ferritin was incubated with CPZ. This observation would be in agreement with Pereira et al. (20), who identified a clathrin-mediated endocytosis pathway for the iron core.

Forms of ferrous iron are widely used as oral supplements due to their relatively high bioavailability. However, they are also associated with side effects such as gastrointestinal intolerance, which can result in noncompliance (35, 36). Oxidative stress generated by ferrous iron salts has been proposed as one of the main reasons for gastrointestinal intolerance (35, 36). We showed that native pea ferritin produced less ROS than FeSO_4_ in equimolar iron concentration. Taking into consideration the relatively high bioavailability and low cost of production, pea ferritin shows promise as a food supplement. There is recent evidence that interactions of recombinant soybean ferritin with some phenolic acids (e.g., gallic acid and its derivatives) inhibit its degradation during simulated digestion (37). Therefore, further research is warranted to explore potential alternatives to deliver in vivo this protein in its native conformation in the intestinal lumen as a novel food supplement.

In conclusion, we report new data relating to the iron bioavailability of a purified ferritin extracted from marrowfat peas. We showed that native pea ferritin is taken up by Caco-2 cells via clathrin-mediated endocytosis and displays lower ROS potential than FeSO_4_. Ensuring the stability of pea ferritin by protecting it from a low gastric pH could generate a novel alternative dietary iron source for the treatment of iron deficiency.

## Supplementary Material

Supplement Figures

## Notes

Supported by HarvestPlus (project code R22850), Biotechnology and Biological Sciences Research Council–Diet and Health Research Industry Club 2 grants (BB/L025396/1 and BB/L025515/1), and a University of East Anglia (UEA) International Studentship (2013–2016).

Author disclosures: AP, IR-R, JR-C, PS, JB, and SF-T, no conflicts of interest.

Supplemental Figures 1–6 are available from the “Supplementary data” link in the online posting of the article and from the same link in the online table of contents at https://academic.oup.com/jn/.

Abbreviations used:

AA: ascorbic acid
BPDS: bathophenanthroline disulfide
CPZ: chlorpromazine hydrochloride
DMT-1: divalent metal ion transporter 1
FAC: ferric ammonium citrate
ROS: reactive oxygen species
siRNA: small interfering RNA
*SLC11A2*: solute carrier family 11 member 2
