# Supplementary material for: Pea Ferritin Stability under Gastric pH Conditions Determines the Mechanism of Iron Uptake in Caco-2 Cells
Source: J Nutr. 2018 Jun 22;148(8):1229–35. doi: 10.1093/jn/nxy096 (PMC6074850; doi:10.1093/jn/nxy096)
Supplement: Supplement Figures [file nxy096_supplement_figures.docx]

**Online Supporting Material**

Supplemental Figure 1**.** Representative Coomassie-stained SDS-PAGE (A) of crude pea ferritin (CF) after salt precipitation and purified pea ferritin (FP) after gel filtration and Western blot (B) of purified ferritin from peas (*Pisum sativum*) stained with Ponceau-S for total protein (left panel) and labeled with anti-pea ferritin antibodies (right panel).

**Online Supporting Material**

**Supplemental Figure 2.** Evaluation of the potential cross-reaction of pea ferritin in human ferritin ELISA kits. Data are means ± SD’s, n = 2. Labeled means without a common letter differ, *P* < 0.05.

**Online Supporting Material**

**Supplemental Figure 3.** Effect of iron concentration (30-500 µM of Fe) from FAC (B) or pea ferritin (C) on Caco-2 cell viability; Triton X-100 (A) used as a positive control of cell death and analyzed by student’s t-test. Data values are means ± SEMs, n = 2 independent experiments. Labeled means without a common letter differ, *P* < 0.05.

**Online Supporting Material**

**Supplemental Figure 4.** Representative NATIVE-PAGE (A,B) of pea ferritin degradation with time at pH 2 without pepsin (A), with pepsin (B), and SDS-PAGE (C) with pepsin.

**Online Supporting Material**

**Supplemental Figure 5.** Estimated distribution of iron (% soluble, protein-sequestered and core iron percentage fractions) at pH 4 (A) and pH 2 (B) for 30, 60, and 120 min. Data are means ± SEMs, n = 2 independent experiments. Labeled means without a common letter differ, *P* < 0.05.

**Online Supporting Material**

**Supplemental Figure 6.** Effect of an Fe^2+^ chelator (BPDS) on iron uptake in Caco-2 cells exposed to native (A) or gastric pH-treated (B) pea ferritin or FAC for 24 h. Data are means ± SEMs, n = 2 independent experiments. Labeled means without a common letter differ, *P* < 0.05.
